# Supplementary material for: Multiparametric Sensing of Outer Membrane Vesicle-Derived Supported Lipid Bilayers Demonstrates the Specificity of Bacteriophage Interactions
Source: ACS Biomater Sci Eng. 2023 May 3;9(6):3632–42. doi: 10.1021/acsbiomaterials.3c00021 (PMC10265573; doi:10.1021/acsbiomaterials.3c00021)
Supplement: Supplementary file 1 — ab3c00021_si_001.pdf [file ab3c00021_si_001.pdf]

Supplementary Material:

Multiparametric sensing of outer membrane vesicle-derived supported lipid bilayers demonstrates the specificity of bacteriophage interactions

Karan Bali[1], Reece McCoy[1], Zixuan Lu[1], Jeremy Treiber[2], Achilleas Savva[1], Clemens F. Kaminski[1], George Salmond[3], Alberto Salleo[2], Ioanna Mela[4], Rita Monson[3], Róisín M. Owens[1]*

1 Department of Chemical Engineering and Biotechnology, Philippa Fawcett Drive, University of Cambridge, Cambridge, CB3 0AS, United Kingdom

2 Department of Materials Science and Engineering, Stanford University, Stanford, California 94305, USA

3 Department of Biochemistry, Hopkins Building, Downing Site, Tennis Court Road, Cambridge, CB2 1QW, United Kingdom

4 Department of Pharmacology, Tennis Court Road, University of Cambridge, Cambridge, CB2 1PD, United Kingdom

7 pages, 7 figures (S1-S7)

SUPPLEMENTARY MATERIAL

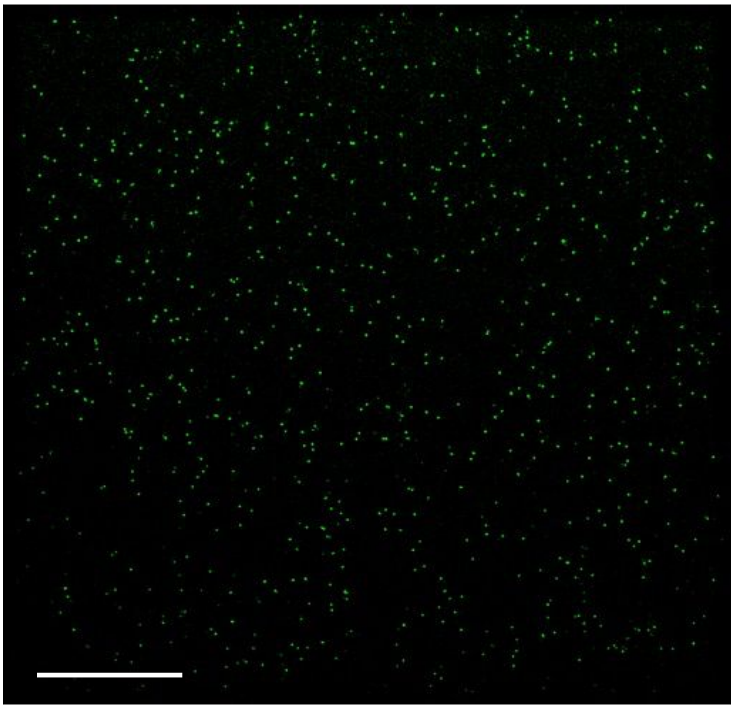

Figure S1. SIM imaging of SYBR green stained T4 phage on a glass slide. Scale bar = 10 μm.

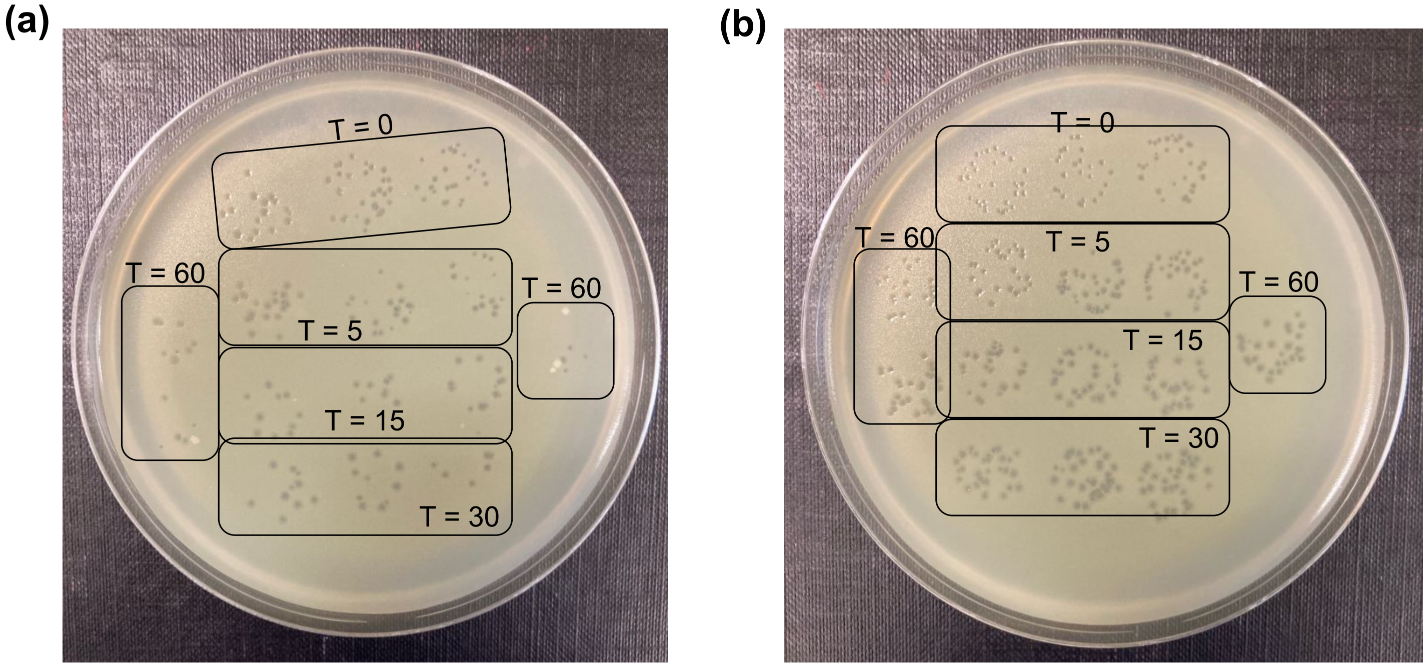

Figure S2. T4 adsorption assay. (a) Plate showing plaques (in triplicates) for phage incubated with overnight *E. coli* culture. (b) Plate showing plaques (in triplicates) for the 'no bacteria' control, where phage was added to LB. Time points are shown in minutes.

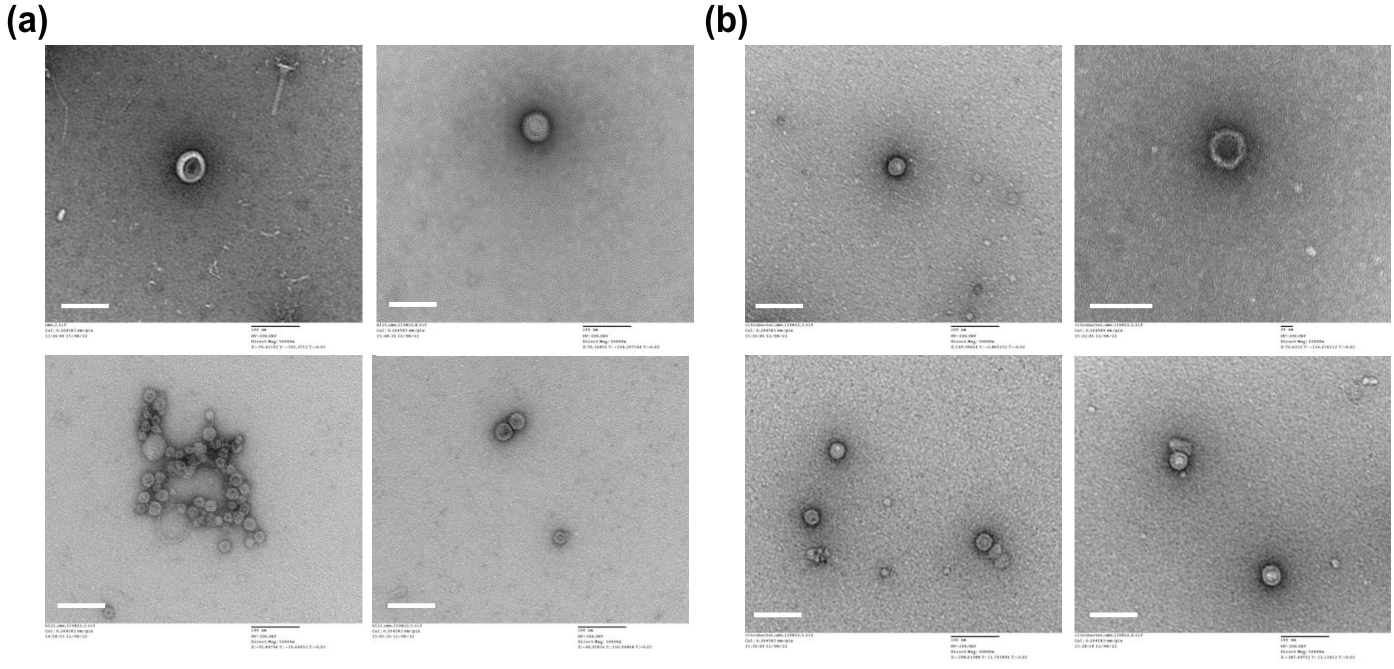

Figure S3. TEM images of OMVs from (a) *E. coli* and (b) *C. rodentium* cells. Scale bars = 100 nm.

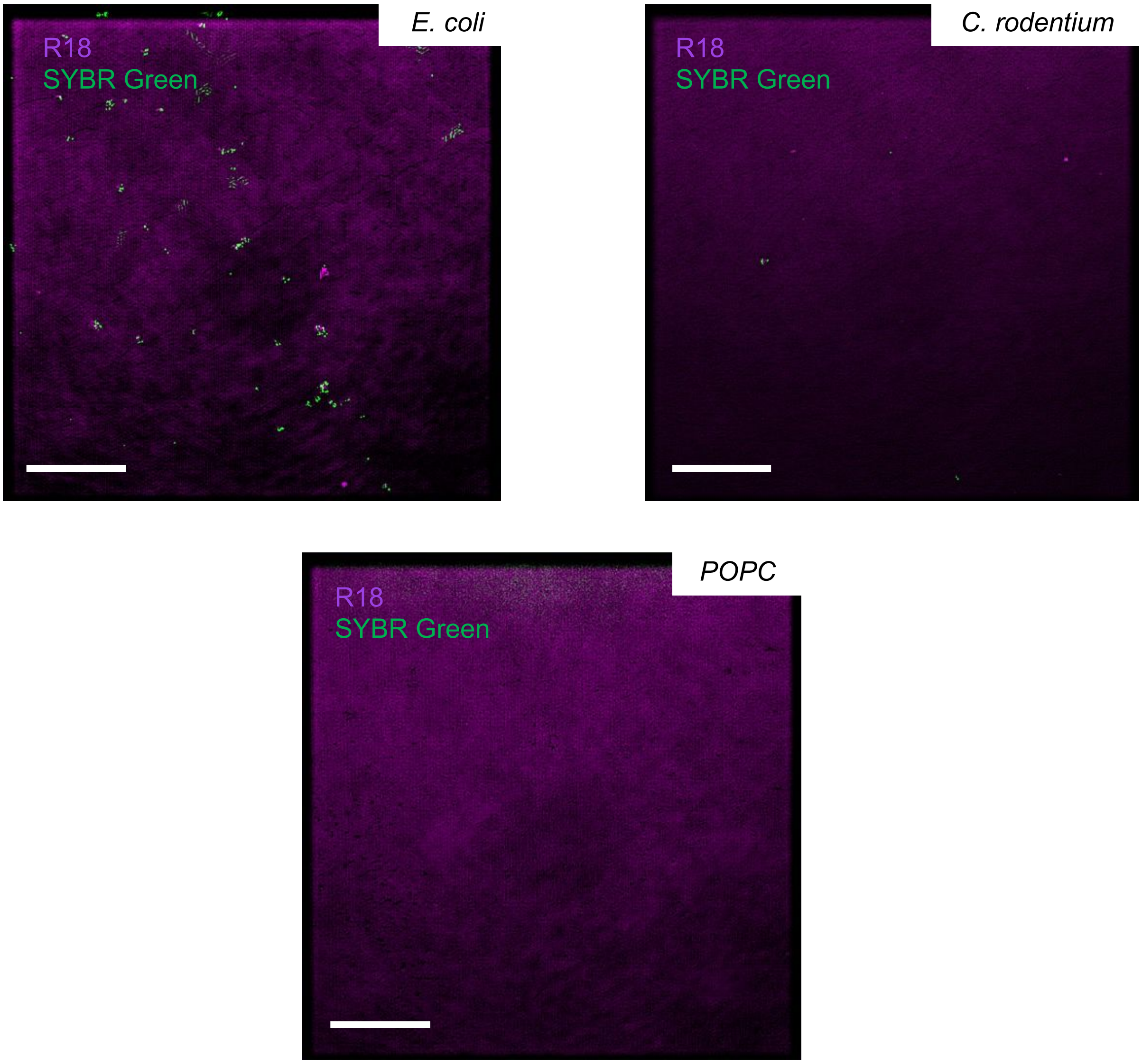

Figure S4. SIM images of the three types of SLBs (*E. coli*, *C. rodentium*, POPC), stained with R18, incubated with SYBR green T4 phage. The areas of green fluorescence in the BL21 SLB are indicative of phage binding. Scale bar = 10 µm.

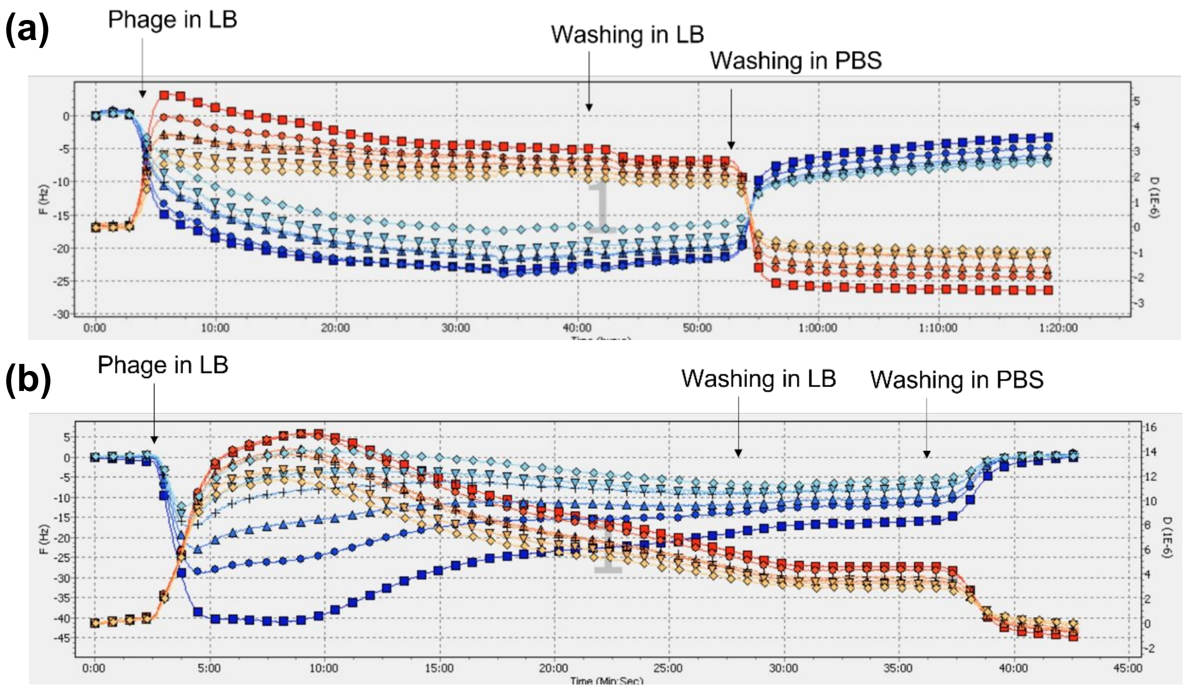

Figure S5. QCM-D graphs showing phage adsorption on (a) OMVs and (b) POPC liposomes on bare gold sensors.

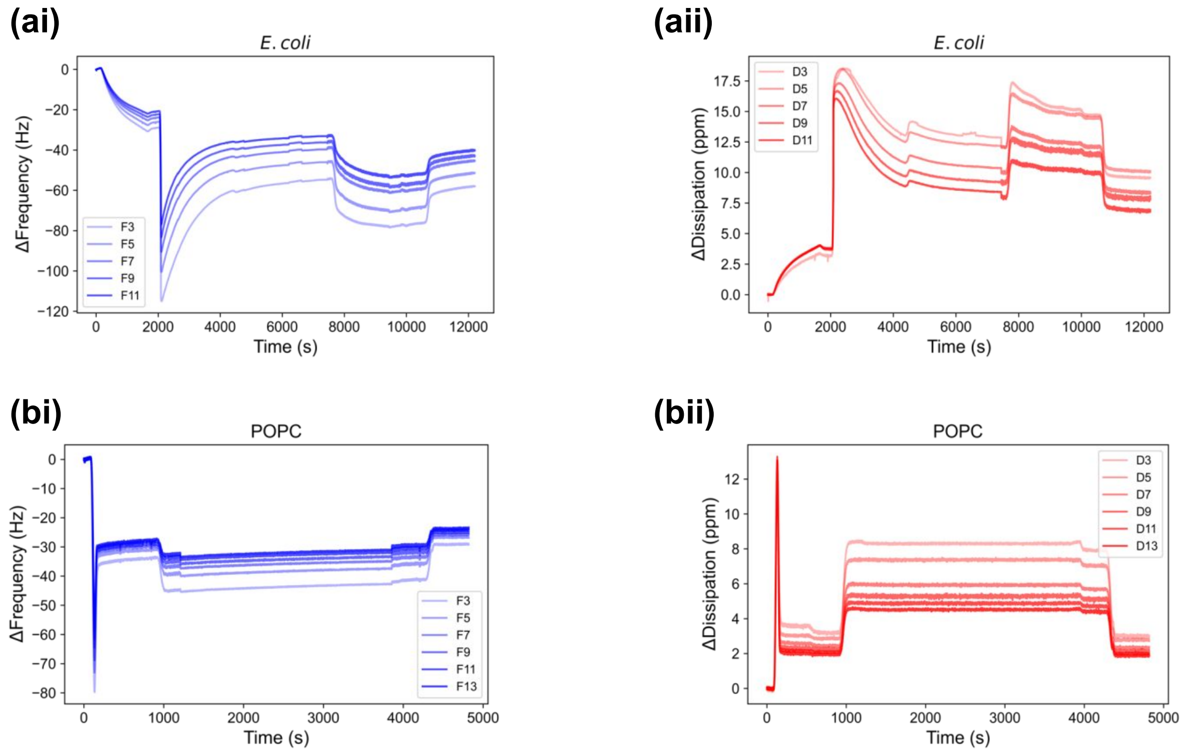

Figure S6. QCM-measurements on SiO_2 sensors. (ai) Δf and (aii) ΔD over time for the *E. coli* SLB and phage addition (added at ~ 8000 s, wash step at ~ 10600 s). (bi) Δf and (bii) ΔD over time for the POPC SLB and phage addition (added at ~ 1000 s, wash step at ~ 4300 s).

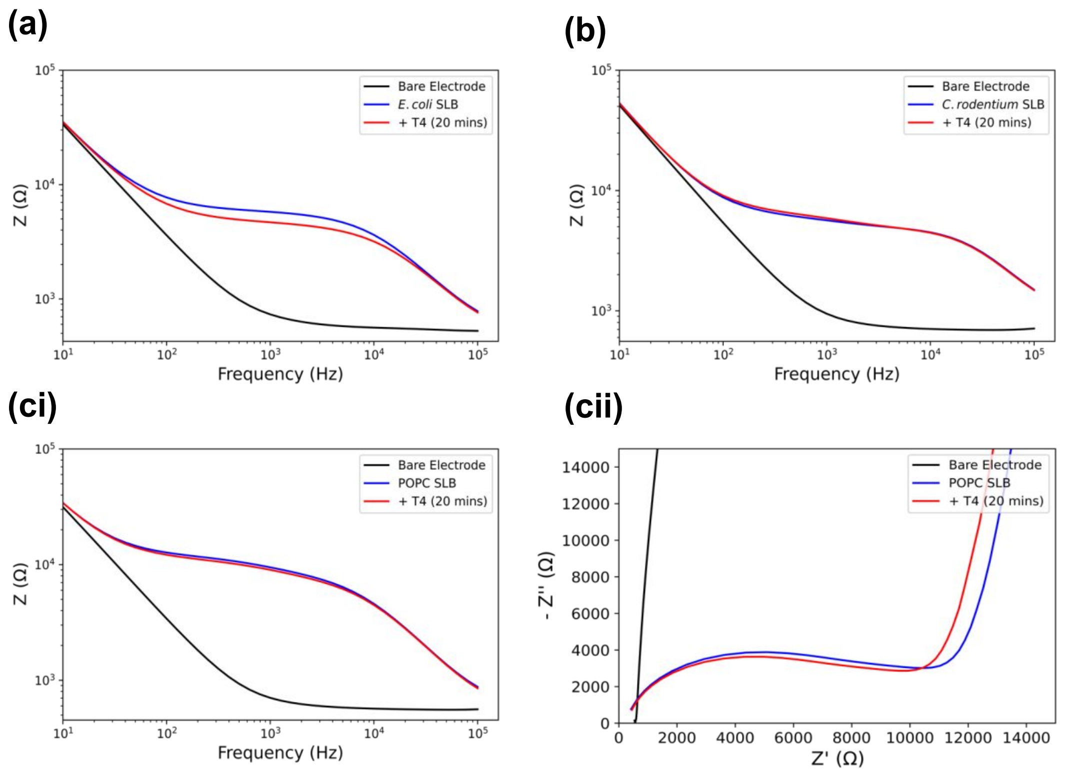

Figure S7. EIS measurements for T4 phage addition to SLBs. Bode plots for (a) *E. coli* and (b) *C. rodentium* SLBs from a representative electrode, corresponding to the Nyquist plots in the main text (figure 4). (ci) Bode and (cii) Nyquist plots from a representative electrode recording for a POPC SLB before and after T4 phage addition.
